# Hair Transplantation on the Baldness Region with Free Latissimus Dorsi Flap for Scalp Reconstruction: A Case Report

**DOI:** 10.1055/s-0044-1787186

**Published:** 2024-06-10

**Authors:** Man Wong Han, Jaeseong Moh, Ji-Ung Park

**Affiliations:** 1 Department of Plastic and Reconstructive Surgery, Seoul National University Boramae Hospital, Seoul National University College of Medicine, Seoul, Republic of Korea; 2 Dr. Moh Hair Transplantation Center, Seoul, Republic of Korea

**Keywords:** baldness, scalp, free flap, reconstructive surgery

## Abstract

Scalp reconstruction, particularly with complex defects and infection risks, often favors microvascular free flaps. However, this method can result in unavoidable alopecia and undesirable aesthetics. This report describes a novel case where hair transplantation via follicular unit extraction (FUE) was applied to a free myocutaneous flap. A 44-year-old woman with Moyamoya disease suffered intracerebral hemorrhage a decade ago. Craniotomies and autologous bone cranioplasties led to wound dehiscence, with subsequent failed local flaps and skin grafts, and identification of a methicillin-resistant *Staphylococcus aureus* infection. The final scalp defect, measuring 13 × 9 cm, was reconstructed using a free myocutaneous latissimus dorsi flap. Nine years post-surgery, a 1,500-unit FUE hair transplantation procedure was conducted. The transplanted hair exhibited robust survival with adequate blood supply, achieving a satisfactory 80 to 85% survival rate at 12 months. This resulted in a notable improvement in the patient's external alopecia, with reported high levels of satisfaction. Free flaps offer a valuable method for scalp defect reconstruction; however, they may not ensure optimal aesthetic satisfaction due to alopecia. Nonetheless, successful FUE hair transplantation on a myocutaneous free flap can yield satisfactory aesthetic results.

## Introduction

Reconstruction of a large scalp defect is traditionally considered highly challenging owing to the convex shape of the scalp, restricted laxity, and the need to achieve satisfactory aesthetic outcomes. 1 2 Scalp reconstruction methods encompass primary closure, skin grafting, local flap reconstruction, and microvascular free flap reconstruction. Notably, the microvascular free flap stands out as a crucial tool for reconstructive surgeons, recognized for its reliability and effectiveness, particularly in addressing extensive and profound scalp defects and deep surgical site infections like osteomyelitis. 3 4

The most commonly used microvascular free flap for scalp reconstruction is the latissimus dorsi (LD) flap, which is divided into myocutaneous and skin-grafted myofascial flaps. A significant drawback of microvascular free flaps is inevitable alopecia, resulting in aesthetically undesirable outcomes.

Hair is important for the social and psychological well-being of individuals, exerting a significant influence irrespective of the patient's sex or age. 5 However, in scalp reconstruction, surgeons often overlook the resulting alopecia, concentrating primarily on the reconstruction. Recognized as an effective adjunctive strategy, hair transplantation plays a pivotal role in harmonizing the skin paddle region of microvascular free flaps. Using follicular unit (FU) micrografts and minigrafts has yielded successful outcomes nearing 90%, thereby improving the aesthetic results of scalp reconstruction surgeries. 6 7

To the best of our knowledge, there is limited documentation regarding the implementation of hair transplantation via follicular unit extraction (FUE) on myocutaneous free flaps. We present a case with aesthetically pleasing results achieved through FUE after reconstructing a scalp defect using a free myocutaneous LD flap. Through this case presentation, we aim to reevaluate and discuss the significant adjuvant management of hair transplantation following microvascular free flap reconstruction.

## Case

A 44-year-old female patient presented with Moyamoya disease and experienced right temporal intracerebral and intraventricular hemorrhages. Following external ventricular drain insertion, craniotomy, and intracranial hematoma evacuation, the patient underwent encephaloduroarteriosynangiosis (EDAS) and cranioplasty with autologous bone in the right parietotemporal area 1 month later.

**Fig. 1:**
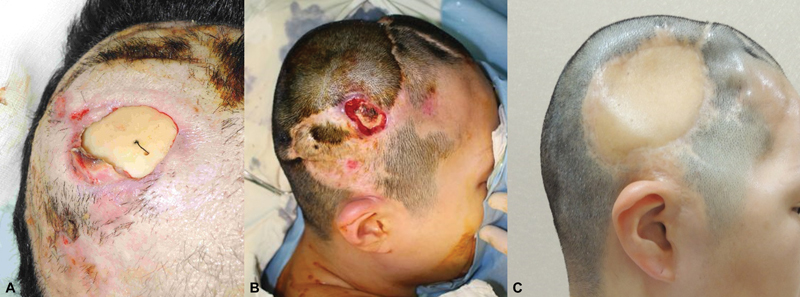
Serial photographs from when the scalp defect occurred to the free flap surgery. ( **A** ) Photograph of wound dehiscence 2 months after craniotomy and cranioplasty due to cerebral hemorrhage. ( **B** ) Persistent wound dehiscence in a local flap and skin graft, a preoperative photograph of the free flap. ( **C** ) Photograph before cranioplasty at 1 year and 11 months after free flap surgery.

After the cranioplasty surgery, a referral for reconstruction was made due to a 2 × 2-cm scalp defect 2 months postsurgery ( Fig. 1A ). Despite attempts with local flaps and skin grafts, there was recurrent wound dehiscence and pus discharge, and methicillin-resistant *Staphylococcus aureus* (MRSA) was identified ( Fig. 1B ). Considering risks related to parietotemporal scalp skin thinning and uncontrolled infection, we decided to perform reconstruction using a microvascular free flap.

The final scalp defect, approximately 13 × 9 cm, was reconstructed using a myocutaneous LD flap from the ipsilateral side. An EDAS procedure utilized the right superficial temporal artery, requiring end-to-end anastomosis of the thoracodorsal artery and vein with the posterior branch of the deep temporal artery and superficial temporal vein, respectively. Postoperative recovery was complication-free, and the infection was resolved.

Eleven months after the flap surgery, pinhole dehiscence at the superior margin resulted in a 1 × 1-cm defect. Screw and plate removal, bone burring, and cranioplasty with a titanium plate were performed. Recurrence of wound dehiscence and MRSA infection led to autologous bone flap removal and subsequent cranioplasty with a titanium plate ( Fig. 1C ). Five months later, a silicone implant corrected a depression in the right temple, and after 4 years, a fat graft further improved the depression in the temple.

**Fig. 2:**
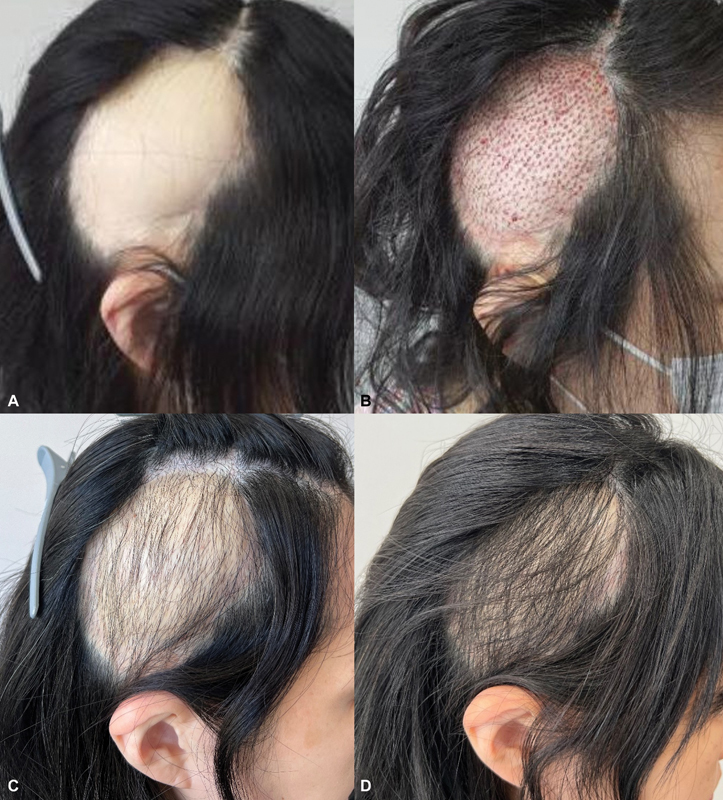
Photographs before and after follicular unit extraction hair transplantation. ( **A** ) Three years after the last scalp surgery, appearance is unsatisfactory due to the baldness region even with long hair. ( **B** ) Immediate postoperative photograph of 1,500 follicular units hair transplantation. ( **C** ) Six-month follow-up photography. ( **D** ) The final graft survival rate at the 12-month follow-up is 80 to 85%.

With the scalp deformity considered adequately addressed, a hair transplant was planned to enhance the aesthetic result of the baldness region with a free LD flap ( Fig. 2A ). The patient's desire for hair transplantation postflap surgery was initially delayed due to wound problems but proceeded after confirming stability and absence of inflammation, occurring 9 years postflap surgery and 3 years after the last scalp surgery.

Before hair transplantation, the baldness region measured 50 cm ^2^ , with a circular shape. Employing the FUE method, 1,500 FUs were harvested from the occipital scalp and transplanted at a density of approximately 30 FUs/cm ^2^ in a single session. The FU harvest was performed without shaving, and serum injections such as platelet-rich plasma were not administered. The recipient area included the skin paddle of the free myocutaneous LD flap and surrounding scars ( Fig. 2B ). After 12 months, the graft survival rate reached 80 to 85%, with no complications observed, showing cosmetic improvement. The patient was satisfied with the postoperative results ( Fig. 2D ).

## Discussion

In cases of cerebral revascularization surgery for Moyamoya disease, decreased scalp perfusion may lead to wound complications and infections. 8 Scalp necrosis was reported in 2 of 112 cases by Houkin et al. 9 Araki et al 8 observed that 14.3% (28 of 195 operations) of cases involved minor events successfully managed with dressing or sutures due to partial necrosis after revascularization, whereas 5.1% (10 of 195 operations) required local flaps or skin grafts due to full-thickness necrosis. Scalp defects from Moyamoya disease pose challenges in selecting recipient vessels for free flaps due to superficial temporal artery sacrifice and potential risks with prolonged surgical procedures. Consequently, local flaps or skin grafts are considered initial treatment options if feasible. 10 In the present case, despite attempts with local flaps and skin grafts for scalp defects, recurring complications and concurrent osteomyelitis necessitated the use of a free flap. The creation of a balanced region by the free flap made it essential to consider hair transplantation from the reconstruction stage onwards. The tissue expander is commonly utilized in the treatment of alopecia; however, the patient, being a young woman, the patient declined its use due to discomfort from prolonged insertion and concerns about configuration deformity. Additionally, because of the substantial size of the flap, relying solely on expanders for complete removal of the skin paddle and covering the entire area with a titanium plate was deemed impractical. Consequently, hair transplantation was prioritized.

During free tissue transfer for scalp defect reconstruction, donor sites include the LD, rectus abdominis, and anterolateral thigh flaps. Among these, the LD flap is an excellent choice for large scalp defects. 11 The LD flap can be myocutaneous or skin-grafted myofascial, with a preference for the latter due to the inhibitory effect of bulky subcutaneous fat on achieving the desired scalp contour. 12 However, skin-grafted myofascial flaps may be disadvantageous for hair transplantation. In FU grafting, hair follicles are transplanted to the midlevel of the recipient dermis, whereas harvested split-thickness skin grafting involves only a portion of the dermis. Although no histological analysis has been conducted for FU grafting with a skin-grafted myofascial flap, studies on FU grafting on a dermal regeneration template (Integra LifeSciences, Plainsboro, NJ) and a skin graft showed hair follicles within the Integra-formed neodermis. 13 Similarly, with a skin-grafted myofascial flap, FUs may not be uniformly transplanted onto the skin graft and could irregularly extend beyond it to the muscle level. Varying depths of hair follicle insertion compromise the consistency of hair growth direction, potentially leading to aesthetically unfavorable outcomes. Further studies are necessary to validate the aesthetic outcome of hair transplantation with a myocutaneous flap versus a skin-grafted myofascial flap.

According to a previous study, patients with a normal scalp have an average survival rate of approximately 90%. 14 Yoo et al 5 reported a survival rate ranging from 70 to 90% (average, 80.67%) in 15 cases of FU grafting on scars from burns or surgery, which is lower than the rate on a normal scalp. Previous studies have only a few publications documenting hair transplantation on free flaps, encompassing both myocutaneous flaps and skin-grafted fascial flaps. 6 7 15 16 17 Transplantation sites have varied, spanning not only the scalp but also regions such as the chin, eyelid, and lower extremities. Survival rates have been reported solely in the study by Blackwell and Rawnsley, 6 observing a relatively good survival rate for skin-grafted myofascial flaps. This case, using the FUE method on a myocutaneous flap demonstrated a survival rate of 80 to 85%. Although <90%, it remains clinically significant, suggesting that FU transplantation for free flaps is worthwhile.

Determining the optimal timing for hair transplantation following microvascular free flap reconstruction of the hair-bearing scalp is crucial, considering infection recurrence and additional procedures for scalp deformities. In this case, due to the patient's history of recurring inflammation, hair transplantation proceeded after confirming stability and absence of inflammation, occurring 9 years after the flap surgery and 3 years after the last scalp surgery. The craniotomy infection rate ranges from 1.1 to 8.1%. 18 Depending on the cranioplasty material, Tokoro et al 19 reported infection onset between 7 days to 42 months (average, 7 months) in 14 cases, with rare infections up to 10 years later. 20 Baumeister et al 15 prioritize soft tissue defect reconstruction in infected cranial bone defects, proposing a 6- to 12-month waiting period before cranioplasty. To avoid infection recurrence, waiting for at least 1 year and ideally longer, with no evidence of infection, is advisable before considering hair transplantation.

When considering the timing for hair transplantation, it is crucial to check if other scalp surgeries are planned. Procedures to correct deformities often include multiple interventions. In this case, silicone implant insertion and fat grafting corrected temple depression. Grafting onto the free flap's skin paddle and scars enhances the camouflage effect during hair transplantation. If additional surgery includes incisions around the flap area, delaying hair transplantation may be effective.

Harvesting methods, including the traditional occipital strip harvesting method known as the follicular unit transplantation (FUT) method and the FUE method, introduced by Rassman et al in 2002, 21 are widely employed in hair transplantation. FUE offers the advantages of less visible scarring and can be performed on a tight scalp. However, FUE is time-consuming, has a longer learning curve, and may result in spot scarring in wider donor areas. 22 In scalp reconstruction with free flaps, where repeated surgeries often lead to scalp tightness, FUE becomes a more appropriate harvesting technique, compared with FUT.

In this case, the hair transplantation density was approximately 30 FU/cm ^2^ . Although ongoing debate exists, suggestions indicate that transplanting hair at a density exceeding 35 FUs/cm ^2^ could potentially decrease graft survival rates and lead to skin necrosis. 21 If skin necrosis occurs, additional reconstruction may be necessary, increasing the risk of infection recurrence. This emphasizes the importance of carefully assessing the vascular supply of flaps and scars before hair transplantation.

The microvascular free flap, a useful reconstruction method for scalp defects, may not provide perfect aesthetic satisfaction due to alopecia. However, satisfactory aesthetic results can be achieved by successfully performing hair transplantation through FUE on a myocutaneous free flap.
